# The Reform of Medical Teaching

**Published:** 1920-07-03

**Authors:** 


### The Hospital

The Workers' Newspaper of Administrative Medicine and Institutional Life, Administration, National Insurance and Health.

No. 1778, Vol. LXVIII. SATURDAY, JULY 3, 1920. PRICE TWOPENCE

### THE REFORM OF MEDICAL TEACHING.

The full report of the Education Committee of the General Medical Council has by this time come to the hands of all the authorities on medical education, and, in that it has been made with special reference to the preventive aspects of medicine, it is more than interesting to realise exactly where we now stand. This week at Cambridge we have the satisfaction of learning that the reforms proposed long ago, and which have been repeatedly promised, may now actually begin to take definite shape.

Two points strike us, however, concerning the various proposals at present advanced. In the first place, it is quite impossible to expect that any moderate revision of the medical curriculum will settle once and for all the problems of medical education. The same problems which face the profession to-day will recur again and again, since medicine promises to take its place among the most progressive of sciences, and those, therefore, who live by its practice will be faced by an ever-extending academic task. The greatest danger of recent movements in the medical schools has come, we think, from a tendency towards over-elaboration of teaching methods. In some quarters we have seen complicated "firms" arranged. In others, one has met interlocking technical courses. Sometimes the whole has been inextricably mixed with undoubtedly valuable attempts at clinical discussions and research meetings. But for the embryo doctor, with the already crowded years of study to be faced, we doubt whether our medical schools will produce a higher average efficiency by these methods of extending indefinitely the methods of twenty years ago, when time and opportunity were more plentiful and the press of personal numbers was less. From this criticism we exclude, of course, the newly instituted unit system, which is to our mind a *reorganisation* of the best type, and, with its full-time directors, likely to produce invaluable results. The new medical era calls for entirely new methods. An outside view of the *elaboration* policy, an inspection of its working schedule, may give one the impression of a perfect hive of industry and a home of ideal academic thought, but, in practice, these things have been but poorly approached. It is true that little time has elapsed since the war, wherein we can judge the results of such first attempts at reform, but it already seems certain to us that those who attempt infinitely to extend the plans of thirty years ago are foredoomed to failure. There is a wholesale and ruthless pruning necessary in the methods of our medical schools, and it is also to be hoped that there will be more standardisation of method among the various teaching bodies concerned. Above all, we must select what is best for the general practitioner's mental equipment, and then by all possible means simplify the methods of his teaching. If he has ambitions for higher professional spheres, that training can come later, and he must be prepared to spend the extra time.

The second phase to claim attention in the Report is the definition of exactly what is indicated by "preventive medicine." Finally and satisfactorily distinction is drawn between this subject and "hygiene and public health." A vast amount of confusion has been raging round the use of these terms, and it will be well if the general Press again gives space to public explanation of the facts, and this time in the light of the Committee's well-considered decisions. Let us quote again from Sir George Newman's memorandum:—

"The proposal is not to add a new subject, nor to lengthen the student's curriculum as a whole, nor even to inflate the subject of hygiene; the essential point is that every medical student, before or after graduation, shall receive proper instruction in these matters (some of which are at present almost wholly ignored), and become imbued with the purpose, practice, and spirit of preventive medicine." It is the preventive aspect of medicine, surgery, and obstetrics as such which must be thoroughly understood. The recommendations made are designed for the ordinary medical student, and not for the student reading for a diploma in public health, and the changes involved comprise an alteration in the type of teaching and in the teacher's own outlook on the subject, but they do not require any special and separate time to be devoted to a "new subject." In any case, since thirty out of thirty-eight of the educational bodies in Great Britain replied to its *questionnaire*, the Education Committee of the Council has been able not only to report in the light of broad views and a high degree of general agreement, but it may therefore in many senses be said to have received representative authority—at last to initiate a series of much-needed changes.

